# DNA methylation of tumor associated calcium signal transducer 2 (*TACSTD2*) loci shows association with clinically aggressive renal cell cancers

**DOI:** 10.1186/s12885-021-08172-1

**Published:** 2021-04-21

**Authors:** Olga Katzendorn, Inga Peters, Natalia Dubrowinskaja, Hossein Tezval, Pouriya Faraj Tabrizi, Christoph A. von Klot, Jörg Hennenlotter, Marcel Lafos, Markus A. Kuczyk, Jürgen Serth

**Affiliations:** 1 Department of Urology and Urologic Oncology, Hannover Medical School, 30625 Hannover, Germany; 2 Department of Urology, Eberhard Karls University of Tuebingen, Tuebingen, Germany; 3 Department of Pathology, Hannover Medical School, Hannover, Germany

**Keywords:** TACSTD2, DNA methylation, Renal cell carcinoma, Prognosis, Survival

## Abstract

**Background:**

DNA methylation is frequently observed in the development and progression of many human tumors as well as renal cell cancer (RCC). Tumor Associated Calcium Signal Transducer 2 (TACSTD2) participates in cell cycle progression through MAPK signalling pathway activation. Moreover, tumor-specific hypermethylation and association with aggressive cancer characteristics has been found for lung adenocarcinoma, hepatocellular carcinoma and cholangiocarcinoma. Whether *TACSTD2* is tumor specifically hypermethylated in RCC or shows association of methylation with adverse clinicopathological parameters and survival of patients has not been investigated at yet.

**Methods:**

Quantitative methylation-specific PCR (qMSP) analysis of a locus in the intron 1 region of *TACSTD2* gene was carried out in a cross-sectional study of 127 paired RCC and normal samples. In silico analysis of *TACSTD2* methylation in the TCGA Kidney Renal Clear Cell Carcinoma (KIRC) dataset of 280 patients served as validation cohort. Statistical analyses were carried out using the two-sided paired t-test for matched tumor and normal sample comparisons, logistic regression for subgroup comparisons, Cox regression for analysis of recurrence free survival (RFS) and Pearson correlation analysis for correlation of *TACSTD2* methylation and *TACSTD2* mRNA in KIRC data.

**Results:**

Higher methylation levels in RCC were significantly associated with advanced disease (*p* < 0.001), high tumor stage (*p* = 0.003), tumor differentiation (*p* = 0.033) and presence of lymph node (*p* = 0.021) or distant metastases (*p* = 0.008). *TACSTD2* hypermethylation was associated with a shorter RFS of patients and demonstrate statistical independency from clinical parameters as state of metastasis, tumor stage, grade and state of advanced disease. In silico validation using TCGA KIRC data also demonstrated association of *TACSTD2* loci with adverse clinicopathology and shortened RFS of patients. In addition, in silico analyses of TCGA KIRC data showed an inverse correlation between DNA methylation levels of TACSTD2 and mRNA expression.

**Conclusions:**

Our results suggest an association between *TACSTD2* methylation and disease progression and clinical course of RCC.

**Supplementary Information:**

The online version contains supplementary material available at 10.1186/s12885-021-08172-1.

## Background

Renal cell carcinoma (RCC) is observed as the 9th common cancer in men and the 14th common cancer in women with an increasing incidence over the past decade [1]. The most frequent histological subtype of RCC is the clear cell RCC (ccRCC). Currently, prognostic predictions relay on clinical and histopathological characteristics [2]. Pathogenesis of different histological subtypes of RCC has been associated with a number of genetic and epigenetic modifications comprehensively analysed in the *The Cancer Genome Atlas network* (TCGA) study providing also molecular data for alterations observed in ccRCC as well as papillary and chromophobe RCC [3]. About 90% of sporadic ccRCC show mutation or loss of the *Von Hippel–Lindau (VHL)* gene function accompanied with subsequent activation of angiogenesis, cell migration and proliferation via the Hypoxia-inducible factor (HIF)-pathway [4]. However, prospective use of gene mutations to enhanced diagnostic or personalized therapy approaches is uncertain as clinically aggressive cancers reveal an individual subset of gene mutations and individual mutation profiles for large part of tumors showed only comparatively weak associations with prognosis and prediction of ccRCC disease [5]. In contrast, several studies, including the work of our group, have identified a substantial number of hypermethylated genes in RCC which are moreover strongly associated with unfavorable histopathological characteristics and/or poor overall survival (OS) [6–9]. So*,* we found hypermethylation of *SFRP1*, *miR-124-3, GATA5*, *CRHBP, NELL1*,*TBR1* and *NEFH* in RCC and demonstrated statistically significant associations with adverse clinicopathology and clinical outcome [10–16]. Furthermore, recent studies also demonstrated an association between tumor-specific hypermethylation and overall survival (OS) in patients receiving targeted therapy [16, 17]. Moreover, some of these studies included additional functional analyses indicating that statistical association with disease characteristics and functional relevance for marker associated genes might be in concordance; although a systematic evaluation of this assumption has not been carried out yet [7, 13].

Tumor Associated Calcium Signal Transducer 2 (TACSTD2) is a transmembrane glycoprotein which is involved in fetal organogenesis, cell proliferation and cell migration via different molecular pathways [18] and is part of the GA733–2 family. It consists of an extracellular part, a transmembrane domain and a cytoplasmatic tail containing a phosphatidylinositol 4,5-bisphosphate (PIP2)-binding motif and a serine residue which is phosphorylated by protein kinase C (PKC) [19]. *TACSTD2* mediates cell cycle progression through activation of the MAPK signalling pathway which is relevant for molecular targeted therapy in RCC [20, 21].

Various solid tumors such as ovarian, colorectal, gastric, breast, endometrial, prostate and bladder cancer have been found to show increased mRNA and/or protein expression levels of *TACSTD2* when compared to corresponding normal tissues [22–25]. Moreover, higher protein expression levels were associated with a worse OS in gastric cancer, colorectal cancer, ovarian cancer and cervical cancer patients [19, 26–28] and associated with clinicopathological features as lymph node metastases or invasive tumours [19, 25, 27, 28]. Hence, *TACSTD2* overexpression has been suggested as a potential prognosticator for various solid tumors [29, 30]. In contrast, an opposite behaviour has been described for *TACSTD2* mRNA and/or protein expression in cancer tissues in lung adenocarcinoma, head and neck squamous cell cancer (HNSCC) and hepatocellular carcinoma (HCC) when compared to the non-tumoral counterpart [31–33]. Correspondingly, in HCC loss of *TACSTD2* protein expression could be linked to a poor OS, metastatic disease and poor differentiation [33]. Notwithstanding that loss of mRNA expression of *TACSTD2* has been described for RCC [34], to our knowledge, DNA methylation alterations of *TACSTD2* and its association with clinicopathology in RCC have not been investigated so far.

Hypermethylation of *TACSTD2* loci has been described in lung adenocarcinoma [34], HCC [33] and cholangiocarcinoma [35]. Moreover, hypermethylation of *TACSTD2* has been associated in all these tumor entities with subsequent epigenetic silencing [33–35] and, in addition, coincided with adjacent organ invasion, poor differentiation and reduced OS [33]. These findings appear to be in line with earlier results identifying *TACSTD2* as a polycomb-regulated target gene in embryonic stem cells often indicative of DNA hypermethylation in malignant cells [36].

Here, we investigated whether methylation of *TACSTD2* gene loci in RCC associate with clinical parameters of tumor aggressiveness and recurrence free survival (RFS) of patients and identified hypermethylated *TACSTD2* loci as a potential prognosticator for RCC. In silico analyses of the KIRC data indicated epigenetic silencing of *TACSTD2* in RCC and confirmed an association of methylation of *TACSTD2* with clinically aggressive subsets of ccRCC.

## Methods

### Patients’ characteristics and study design

To elucidate a potentially relevant association of the *TACSTD2* loci DNA methylation and the clinical characteristics of patients we analysed a cohort of 122 fresh frozen renal tumor tissues (Table 1) by the use of a cross-sectional study design. For detection of a possible tumor-specific hypermethylation we compared the 122 tumor tissues to corresponding 122 histopathological normal, i.e. tumor adjacent tissue samples. Tissue sampling, pathological tissue assessment, preparation and storage have been described before [15, 37]. A subset of 77 patients with an appropriate follow-up was subjected to Kaplan-Meier and cox regression survival analyses. The study was approved by the ethics committees of the Faculty of Medicine of the Eberhard Karls University Tuebingen (Head Prof. Luft) and Hannover Medical School (Head Prof. Tröger) (ethics votes no. 128/2003 V and 1213–2011) and written informed consent was obtained from patients. The study was carried out in accordance with the Helsinki declaration.

**Table 1:** Patient’s clinical and histopathologic characteristics

		Number of patients, n (%)	Subset with FU, n (%)
**Total**		122 (100.0)	77 (100.0)
**Histologic subtype**	ccRCC	86 (70.5)	57 (74.0)
	pap. RCC	24 (19.7)	17 (22.1)
	chrom. RCC	3 (2.5)	2 (2.6)
	Mixed histology	5 (4.1)	1 (1.3)
	Other	4 (3.3)	0 (0)
**Gender**	Female	43 (35.2)	27 (35.1)
	Male	79 (64.8)	50 (64.9)
**Age (years)**	Median	64.5	65
	Min-Max	35–91	37–91
**Metastasis**	M0	95 (77.9)	59 (76.6)
	M1	27 (22.1)	18 (23.4)
	NA	0 (0.0)	
**Lymph node status**	N0	107 (87.7)	70 (90.9)
	N1	15 (12.3)	7 (9.1)
**Tumor stage**	pT1	11 (9.0)	8 (10.4)
	pT1a	35 (28.7)	24 (31.2)
	pT1b	21 (17.2)	13 (16.9)
	pT2	8 (6.6)	6 (7.8)
	pT3	5 (4.1)	2 (2.6)
	pT3a	12 (9.8)	4 (5.2)
	pT3b	25 (20.5)	18 (23.4)
	pT3c	3 (2.5)	2 (2.6)
	pT4	1 (0.8)	0 (0.0)
	NA	1 (0.8)	0 (0.0)
**Differentiation**	G1	24 (19.7)	14 (18.2)
	G1–2	16 (13.1)	10 (13.0)
	G2	62 (50.8)	42 (54.5)
	G2–3	9 (7.4)	5 (6.5)
	G3	11 (9.0)	6 (7.8)
State of disease*	Localized	65 (53.3)	42 (54.5)
	Advanced	56 (45.9)	35 (45.5)
	NA	1 (0.8)	0 (0.0)

Abbreviations: ccRCC clear cell renal cell carcinoma (RCC); pap. RCC papillary RCC; chrom. RCC chromophobe RCC; FU follow-up; NA not available

*Localized disease defined as pT ≤ 2, N0, M0; Advanced disease defined as pT ≥ 3 and/or N+, M+

### Nucleic acid isolation, bisulfite conversion of DNA and quantitative methylation-specific real-time PCR (qMSP) analysis

DNA was isolated and converted as described before [11]. The qMSP primer system comprised of the forward primer 5′- GAAACCCCGAACCATAATAAAACGA − 3′, the reverse primer 5′- ACGTCGGAGTTCGAGTTTCG − 3′ and the probe 5′-FAM- CGAACCGAACGCGAACGAATAAAACGC -BHQ-3′. All primers were designed by use of the Beacon Designer software (PREMIER Biosoft, Palo Alto, CA, USA). The *TACSD2* – qMSP includes 14 CpG sites on chromosome 1 at positions 59,042,814, ~ 822, ~ 837, ~ 847, ~ 852, ~ 856, ~ 858, ~ 862, ~ 871, ~ 873, ~ 884, ~ 891, ~ 898 and ~ 901 referring to the hg19 genome assembly (Fig. 4a). The qMSP measurement was tested in advance on its PCR efficiency and linearity as described before by others [38]. Control reactions were part of each measurement (suppl. Fig. 2) and calculation of sample specific relative methylation values was carried out as described before [11].

### Statistical analyses

For candidate identification we performed an in silico analyses of the TCGA KIRC dataset. For this purpose, the level 3 data of the TCGA KIRC HM450k methylation data set [39], the statistical software R 3.6.1 and a × 86 64bit desktop computer platform with 32 GB RAM / Windows 7 was used.

We compared methylation of *TACSTD2* loci in kidney tumor tissue and paired adjacent normal kidney tissue using the two-sided paired t-test. *P* value < 0.05 was considered to be statistically significant. Bivariate logistic regression models were performed to compare dichotomized tumor groups for methylation differences considering age as a covariate. Groups were dichotomized depending on clinicopathological characteristics as follows: presence or absence of local or distant metastasis, high and low tumor stage (T) or grade (G) categorization by comparing the T1 - T2 versus T3 - T4 groups and G1-G2 versus G2–3 group, respectively. Odds ratios (OR) and confidence intervals (CI) were given for each calculation. Recurrence free survival was calculated using Cox’s proportional-hazards regression model. The optimum cut off value was approximated for dichotomization of methylation levels with respect to a logrank statistics using R 3.6.1 [40]. and the ‘maxstat’ package [41].

## Results

### Comparison of TACSTD2 methylation in RCC and paired normal kidney tissue samples

Comparison of *TACSTD2* methylation levels in ccRCC and papillary RCC showed no statistically significant difference in mean methylation (*p* = 0.90, OR = 0.99, 95% CI: 0.87–1.13). Significant gender-specific differences in methylation levels were found neither in tumor tissues (*p* = 0.790, OR = 1.01, 95% CI: 0.92–1.13) nor in adjacent histopathological normal tissue samples (*p* = 0.297, OR = 1.10 95% CI: 0.92–1.31). Thus, statistical analyses for association with clinicopathological parameters and survival of patients were carried out for the complete cohort of RCC samples without consideration of histological subtype or gender as covariates.

**Fig. 1:**
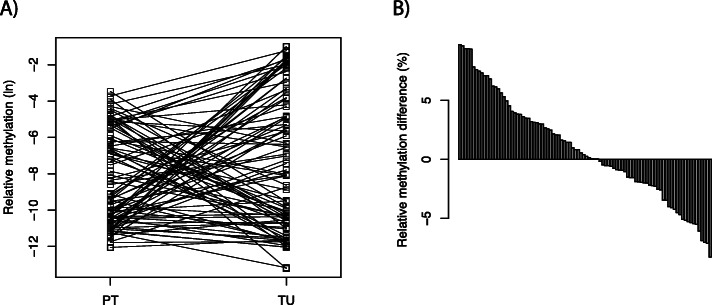
Comparison of *TACSTD2* methylation in paired renal tumor and adjacent normal tissue. **a** Comparison of natural logarithms of relative methylation values in tumor tissue (TU) and adjacent normal tissues (PT). **b** Assorted paired difference plot for pairwise relative methylation differences (%) of *TACSTD2* methylation in paired tumor and adjacent normal tissue

Our comparative methylation analysis of paired tumoral and normal tissues revealed a complex result. The line segments of Fig. 1a, each connecting relative methylation values obtained for the tumor adjacent normal and paired tumoral tissue samples, show subgroups with clear tumor-specific hypermethylation but also a subset with tumor specific hypomethylation. Moreover, a part of tissue pairs did not demonstrate considerable variation in methylation differences, although absolute methylation levels among tissue pairs varied substantially. Using an assorted difference plot it appears that about one third of the tumors presented with hypermethylation, the other thirds demonstrated either no pronounced alteration in methylation levels or even tumor hypomethylation (Fig. 1b). Correspondingly, statistical comparison using the paired t-test revealed statistically no significant difference between tissue pairs (*p* = 0.068, two-sided t-test).

### Comparison of methylation of the TACSTD2 CpG locus and clinicopathological characteristics of tumors

To assess the potential usefulness of *TACSTD2* loci methylation we compared relative methylation values in dichotomized tumor groups using bivariate logistic regression including age as a covariate to control a potential bias due to age related methylation effects.

**Fig. 2:**
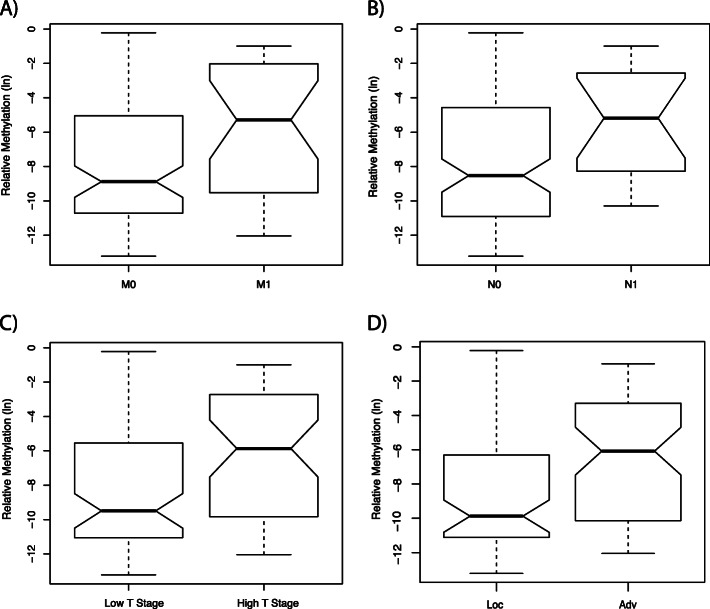
Box plot illustration of *TACSTD2* methylation and its association with clinicopathological characteristics. The box plot illustrates the median, the estimated confidence intervals, and the 25% quartiles in both groups. Presentation of the natural logarithm of relative methylation of metastasis negative (M0) versus metastasis positive (M1) tumors (**a**), lymph node negative (N0) versus lymph node positive (N1) tumors (**b**), low (defined as T1 and T2) versus high (defined as T3 and T4) tumor stage (**c**) and localized (Loc. defined as pT ≤ 2, N0, M0) versus advanced disease (Adv. defined as pT ≥ 3 and/or N1, M1) (**d**). Statistical analyses showed significant higher methylation levels in metastasis positive tumors, high tumor stage and advanced disease (Table 2)

Comparison of 95 patients without distant metastases (M0) and 27 patients with metastatic disease (M1) revealed a significant increase in mean methylation in the M1 group (*p* = 0.008, OR = 1.18, 95% CI: 1.05–1.35;Table 2, Fig. 2a) while age was no significant parameter of the statistical model (*p* = 0.099).

**Table 2:** Statistical association of *TACSTD2* methylation and clinicopathological characteristics in logistic regression analyses

TACSTD2 methylation	OR (95% CI)	p-value
Metastasis (M0 vs. M1)	1.18 (1.05–1.35)	0.008
Lymph node status (N0 vs. N1)	1.21 (1.03–1.43)	0.021
Tumor stage (low vs. high T*)	1.18 (1.06–1.31)	0.003
Differentiation (low vs. high G**)	1.16 (1.01–1.34)	0.033
State of Disease (loc vs. adv***)	1.20 (1.08–1.34)	< 0.001

Abbreviations*:* vs versus

OR odds ratio; 95% CI 95% confidence interval

*Low defined as T1 and T2; high defined as T3 and T4

** Low defined as G1, G2; high defined as ≥G3

***Localized disease (loc) defined as pT ≤ 2, N0, M0; advanced disease (adv) defined as pT ≥ 3 and/or N1, M1

Comparing 75 tumors of low stage (pT1 or pT2) with 46 tumors of high stage disease (pT3 or pT4) demonstrated a significant increase in methylation for the high stage group (*p* = 0.003, OR = 1.18, 95% CI: 1.06–1.31; Table 2, Fig. 2c), but no significant contribution of the covariate age (*p* = 0.844).

The state of lymph nodal disease (107 cases without lymph node metastasis (N0) and 15 cases with one or more positive lymph nodes (N1/2) showed a significant increase in mean methylation (*p* = 0.021, OR = 1.21, 95% CI: 1.03–1.43; Table 2, Fig. 2b). The covariate age also did not reach statistical significance in the bivariate regression model (*p* = 0.153).

Comparison of low grade and high grade tumors showed a significant increase in methylation (*p* = 0.033, OR = 1.16 95% CI: 1.01–1.34; Table 2) without significant contribution to the covariate age (*p* = 0.110).

Moreover, methylation comparison between localized and advanced cancers revealed that tumors classified as advanced disease showed higher mean methylation at *TACSTD2* loci (*p* < 0.001, OR = 1.20, 95% CI: 1.08–1.34; Table 2, Fig. 2d). The covariate age did also not reach statistical significance (*p* = 0,979).

### Association of methylation and recurrence free survival of patients

**Fig. 3:**
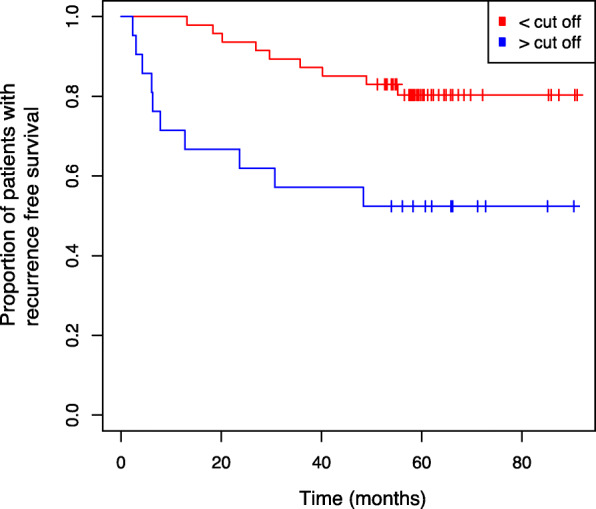
Kaplan-Meier survival analysis of *TACSTD2* methylation and RFS. Kaplan-Meier survival analysis showing RFS of patients with methylation levels above and below of the optimum cut off value determined for relative methylation of − 6.99 (natural logarithm) corresponding to 0.1% relative methylation

To evaluate whether methylation of *TACSTD2* loci is associated with the RFS of patients a subset with available follow-up information was subjected to Kaplan-Meier analysis following determination of the optimum cut point and dichotomization of patients. Using an optimum cut point approximately corresponding to 0.1% relative methylation, patients with primary tumors exhibiting above cut point methylation demonstrated a significant faster disease progression (*p* = 0.005, logrank, Fig. 3). Already after 10 months follow-up period six patients demonstrated progress compared to none of the lower methylated group. However, survival curves did not show further divergence over time but went roughly parallel until the maximum observation period of about 90 months is reached.

In view that a multivariate evaluation of the prognostic relevance of *TACSTD2* methylation might be biased due to the limited size of subgroups, pairwise bivariate cox regressions were carried out as a methodical surrogate to define possible statistical dependencies on clinicopathological parameters. Bivariate cox regression modelling considering state of metastasis, stage, grade and status of advanced disease as covariates revealed methylation as a significant variable indicating *TACSTD2* methylation as an independent prognosticator (Table 3).

**Table 3:** Association of TACSTD2 methylation and clinicopathological parameters with recurrence free survival in bivariate survival analysis

	HR (95% CI)	p-value
TACSTD2 methylation	2.85 (1.14–7.12)	0.025
Metastasis	4.92 (1.95–12.43)	0.001
TACSTD2 methylation	2.85 (1.14–7.16)	0.026
Tumor stage*	2.77 (1.10–6.95)	0.030
TACSTD2 methylation	3.59 (1.42–9.04)	0.007
Differentiation	11.48 (4.13–31.91)	< 0.001
TACSTD2 methylation	2.37 (0.93–5.99)	0.069
State of Disease**	4.72 (1.65–13.50)	0.004
TACSTD2 methylation	3.39 (1.37–8.35)	0.008
Age	0.99 (0.95–1.03)	0.571

HR hazard ratio; 95% CI 95% confidence interval

*Low defined as T1 and T2; high defined as T3 and T4

**Localized disease defined as pT ≤ 2, N0, M0; Advanced disease defined as pT ≥ 3 and/or N1, M1

### In silico re-evaluation of TACSTD2 DNA methylation using the TCGA KIRC data

**Fig. 4:**
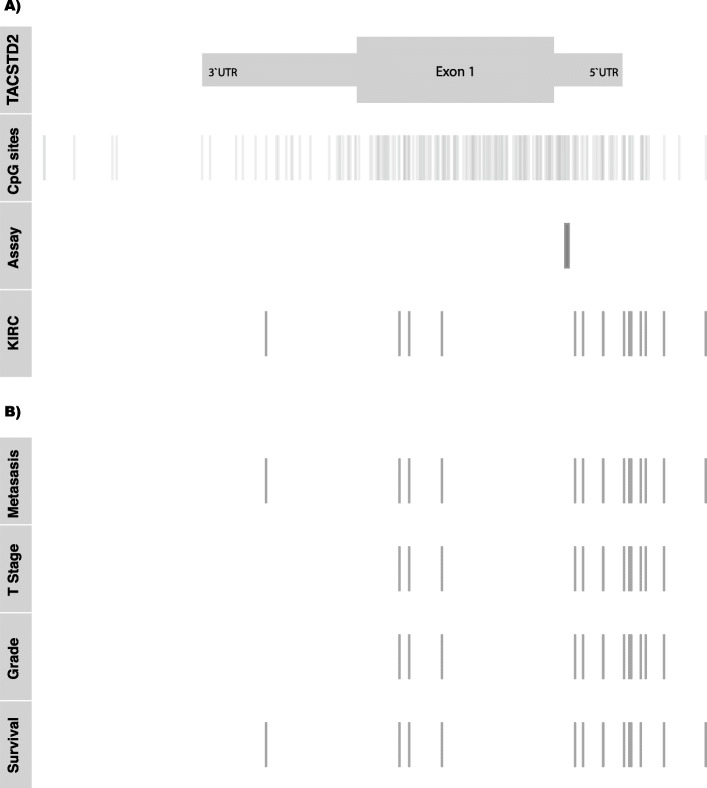
Genomic organization of the *TACSTD2* gene on chromosome 1. **a** Locations for 5’UTR and 3’UTR corresponding genomic regions including exon1 (*TACSTD2*), all CpG sites present in the region (CpG sites), the region covered by the qMSP assay (Assay), and CpG sites annotated for the KIRC study (KIRC). **b** Location of CpG sites showing significant association (*p* < 0.05) with clinicopathological parameters state of distant metastasis, high or low stage status of tumor stage and tumor grade as well as univariate association with RFS in in silico analyses of KIRC data

To independently evaluate our findings for the association of methylation with clinicopathological parameters and RFS we questioned the TCGA-Kidney Renal Clear Cell Carcinoma (KIRC) database. We found 15 CpG sites, annotated to the transcription start sites, a genomic region corresponding to the 5’UTR of *TACSTD2* mRNA, the first exon as well as the 3’UTR corresponding region of the *TACSTD2* gene (Fig. 4, row “KIRC”) providing evaluable data. Twelve of 15 loci were covered by the large CpG island, while three sites were classified as shore-CpG sites. It turned out that 15 out of 15 (100%) of these sites were significantly associated with the state of distant metastasis (Fig. 4, row “Metastasis”, Table 4). Higher methylation of 12 out 15 (80%) loci appeared as significantly associated both with high stage and high grade tumors (Fig. 4, rows “Stage” and “Grade”, Table 4). Loci showing association with metastasis, stage and grade of tumors span over the gene including sites of transcriptional regulation, gene body as well as 3’UTR corresponding region. The results of in silico univariate cox regression survival analysis showed significant association of methylation of loci between the status of metastasis and survival of patients (Fig. 4, rows “Metastasis.” and “Survival.”, Table 4).

**Table 4:** In silico validation of *TACSTD2* methylation results using TCGA KIRC data

Locus	Position on chromosome 1	^**1**^Clinicopathology						^**2**^survival data	
		Metastasis		Tumor stage		Differentiation			
		p	OR	p	OR	p	OR	p	HR
cg21536783	59,041,407	3.10*10^−03^	65.32	2.40*10^−01^	2.58	6.11*10^−01^	1.50	2.97*10^−02^	1.88
cg00667789	59,042,065	3.91*10^−05^	44.44	8.56*10^−05^	14.03	1.34*10^−05^	18.92	2.97*10^−04^	2.50
cg00554413	59,042,113	2.14*10^−04^	104.97	3.34*10^−03^	14.66	1.32*10^− 03^	18.75	3.27*10^− 02^	1.56
cg24851854	59,042,275	5.51*10^−07^	68.88	3.67*10^−08^	39.14	3.39*10^−08^	47.07	4.95*10^−06^	2.83
cg05065507	59,042,931	1.11*10^− 06^	65.79	2.03*10^−05^	31.81	6.40*10^−04^	19.86	7.70*10^− 06^	2.66
cg10347335	59,042,971	4.47*10^− 07^	359.26	2.05*10^−05^	74.78	8.85*10^−04^	32.63	1.54*10^−03^	1.98
cg13443627	59,043,070	8.56*10^−07^	95.42	2.77*10^−06^	28.12	1.07*10^−05^	23.73	4.99*10^−04^	2.05
cg17210938	59,043,173	2.82*10^−05^	116.62	1.05*10^−05^	65.44	1.11*10^−03^	21.10	1.80*10^−05^	2.54
cg16080552	59,043,199	5.62*10^−06^	47.95	3.98*10^−05^	17.08	1.18*10^−04^	15.43	4.76*10^−07^	2.88
cg04863005	59,043,208	5.34*10^−06^	36.00	1.39*10^−05^	14.05	2.96*10^−04^	8.81	1.34*10^−05^	2.66
cg16699148	59,043,255	2.53*10^−04^	54.55	3.32*10^−03^	13.26	1.47*10^−02^	8.66	6.59*10^−03^	1.76
cg01821018	59,043,280	3.04*10^−04^	180.71	1.03*10^−02^	13.66	1.12*10^−02^	12.72	7.23*410^−02^	1.48
cg19813884	59,043,370	8.42*10^−06^	2179.41	2.3*10^−03^	80.46	3.10*10^−02^	23.44	1.61*10^−03^	1.93
cg05346878	59,043,576	8.10*10^−04^	16.72	5.51*10^−01^	1.48	3.58*10^−01^	1.83	2.82*10^−02^	1.93
cg27398499	59,043,873	1.13*10^−04^	27.96	7.97*10^−01^	1.19	4.06*10^−01^	1.76	1.19*10^−01^	1.46

HR Hazard ratio, OR Odds ratio, p p-value, NA not available

1 univariate logistic regression for methylation comparison of dichotomized tumors for detection of statistical association with distant metastasis (M), high (> = T3) and low stage (< T3), as well as low (< G3) and high grade (> = G3) tumor subsets. Please note that two cpg sites could not be analysed

2 cox regression analysis for methylation and recurrence free survival

### Analysis of statistical association between TACSTD2 DNA methylation and mRNA expression

Whether DNA methylation shows association with alteration of mRNA expression was also investigated by use of the KIRC data. In silico analysis revealed that all of the 15 loci described above to be amenable for in silico analysis show a significant inverse relationship between methylation and mRNA expression in linear regression analyses (suppl. Fig. 1). Pearson correlation analysis revealed coefficients of correlation ranging between − 0.69 and − 0.29 (*p* < 0.001, Bonferroni-Hochberg correction for multiple testing).

### Tumor specific loss of TACSTD2 mRNA expression in RCC

**Fig. 5:**
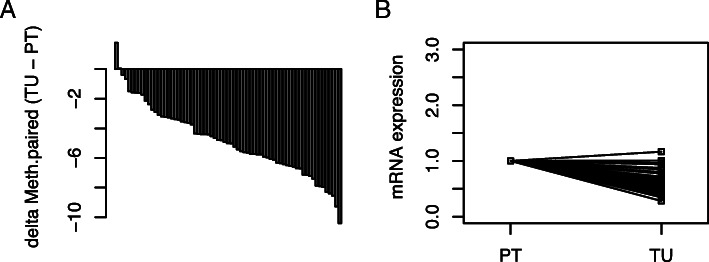
Comparison of *TACSTD2* mRNA in paired renal tumor and adjacent normal tissue. **a** Assorted paired difference plot for pairwise mRNA differences of *TACSTD2* mRNA in paired tumor (TU) and adjacent normal tissue (PT). **b** Comparison of natural logarithms of mRNA values in tumor tissue (TU) and adjacent normal tissue (PT)

We compared *TACSTD2* mRNA expression as reported by the TCGA KIRC data in tumor adjacent normal and paired tumoral tissue and found lower levels of *TACSTD2* mRNA in tumoral tissues (*p* < 0.001, paired t-test, Fig. 5).

### Evaluation of TACSTD2 protein expression in RCC samples using proteinatlas.org

To assess protein expression of TACSTD2 in RCC we interrogated the proteinatlas.org database including immunostainings for the TACSTD2 protein in RCC tissues and non-tumorous kidney tissues with three different antibodies. Two of three antibodies showed immunopositivity in normal kidney tissues in about 25% of tubular epithelial cells in 3 of 3 cases. In contrast, a complete loss of immunopositivity could be observed in 83% (10/12) and in 100% (12/12) RCC samples for both responsive antibodies [42].

## Discussion

Our analyses show that higher methylation of the *TACSTD2* gene in RCC is associated with unfavorable clinicopathological parameters that in general are found in tumors of higher clinical aggressiveness. So, DNA hypermethylation demonstrated statistically significant association with the presence of distant metastasis, one of the strongest prognosticators of RCC. In line, comparison of methylation and clinical stage of tumors, tumor differentiation as well as the status of advanced disease, which are known to be of prognostic value in RCC, also exhibited a significant statistical relationship of increased methylation and adverse clinical status of patients.

In line with a possible relevance of *TACSTD2* methylation as a prognosticator for an unfavorable course of the disease we found that tumors showing increased methylation exhibit a significantly shorter period until tumor recurrence which, of interest, was likely independent from the strong prognosticators state of metastasis or advanced tumor classification.

To gain additional and independent statistical evidence for the association between *TACSTD2* DNA methylation and a worse clinical development of the disease we also carried out in silico analysis of the TCGA KIRC methylation data. Interestingly, we found for the great majority of CpG sites amenable for evaluation a significant relationship of increased methylation of loci and unfavorable clinicopathological parameters as well as decreased RFS of patients. Therefore, two independent cohorts each measured by a different methylation detection method, agree that *TACSTD2* methylation is statistically associated with a clinically more aggressive phenotype of RCC. In concordance with our analysis for RCC, an association of *TACSTD2* hypermethylation and poor OS has been previously reported for patients suffering from aggressive HCC [33].

Our in silico analysis showed a negative correlation of methylation and mRNA expression for all of the investigated 15 CpG sites thus indicating epigenetic silencing of *TACSTD2* in RCC. Hypermethylation and concurrent loss of *TACSTD2* mRNA expression as a potential mark for epigenetic silencing has already been demonstrated for a number of human malignancies such as cholangiocarcinoma [35], lung adenocarcinoma [34], malignant glioma [43] and HCC [33]. Whether the association of increased DNA methylation with worse clinical outcome can be supported by a corresponding finding on the mRNA expression level has been investigated by a literature research of proteinatlas.org. Interestingly, loss of *TACSTD2* mRNA expression turned out to be associated with a shorter recurrence free survival of patients (*p* < 0.001) [42]. Furthermore, a loss of immunopositivity could be found in the majority of investigated RCC tissues compared to normal renal tissue samples [42].

Summing up, our findings of *TACSTD2* DNA methylation alterations in RCC and the corresponding in silico analyses including KIRC data set (methylation, mRNA expression) as well as proteinatlas.org data (protein expression) could point to an association of loss of *TACSTD2* expression with a more aggressive biological phenotype of RCC. Contrary results have been observed in other tumor entities. In gastroenterological and gynecological cancers an increased TACSTD2 protein expression level was associated with adverse clinicopathology and poor outcome [29].

Different findings in various tumor entities nevertheless emphasize the necessity for further detailed analyses of the functional context of *TACSTD2* as well as additional epigenetic mechanism possibly relevant for *TACSTD2* function. In silico analyses indicated homogeneous loss of *TACSTD2* mRNA and protein expression in RCC, although methylation of *TACSTD2* loci in our analysis clearly was heterogeneous. A hypothetical explanation for this contradiction could be the presence of an additional layer of epigenetic regulation of *TACSTD2* mRNA expression. Such is indicated by the UCSC table browser demonstrating a binding site for miR-495 microRNA in the 3′-UTR corresponding *TACSTD2* gene region. miR-495 was previously described to suppress cell proliferation and migration in RCC [44] and epigenetic regulation of miR-495 was linked to tumor suppression in breast cancer [45]. Moreover, post-transcriptional regulation of *TACSTD2* expression by miR-125b as well as promotion of cell migration in RCC has been reported previously [46, 47]. Also, functional in vitro and in vivo analyses for *TACSTD2* in other human tumor entities like lung adenocarcinoma, HCC and cholangiocarcinoma, consistently demonstrated that silencing of *TACSTD2* gene expression results in a significant increase of tumor growth [34] and leads to cell proliferation and migration [33, 35]. Interaction with the Insulin-like growth factor (IGF)-1/IGF-1R axis and ErB3 activation, both known to be involved in oncogenic processes, have been suggested as a possible molecular mechanism [33–35]. Interestingly, high levels of IGF-1/ IGF1-R were also reported to be associated with poor OS and cancer aggressiveness in RCC [48].

## Conclusion

Conclusively, a substantial number of epigenetic alterations were described in RCC in part showing statistically significant association with clinicopathological parameters of patients, but as yet no marker or marker-panel has been transferred into clinical routine [2]. Our analyses identify *TACSTD2* DNA methylation as a new promising candidate marker associated with clinically aggressive RCC. Our results suggest inclusion of *TACSTD2* DNA methylation in corresponding future prospective biomarker candidate panel analyses and for detailed functional analysis in RCC.

## Supplementary Information

**Additional file 1: Suppl. Fig. 1**: Correlation between *TACSTD2* methylation and *TACSTD2* mRNA expression. In silico analysis by Pearson correlation analysis reveal a statistically significant (*p* < 0.05) inverse relationship between *TACSTD2* methylation (MethylVal) and *TACSTD2* mRNA expression (Expr) in all investigated *TACSTD2* loci. The Correlation coefficient (R) and the *p*-value are specified for each locus.

**Additional file 2: Suppl. Fig. 2**: Exemplary primary data of quantitative methylation specific real-time PCR (qMSP). Sixteen measurements are required for determination and quality control of a single sample methylation. Measurements, presented without base-line adjustement, were carried out using either the Alu-C4 probe for adjusting for the input amount of converted DNA in blank, 0% methylation, 100% methylation and sample measurements each in duplicate (Group A) or aliquots of samples for corresponding measurements by use of the TACSTD2 probe (Group B). Note, that Alu-C4 detects a repetitive sequence motif resulting in considerable lower Ct values as compared to the single copy target sequence detection.

## Data Availability

The anonymized datasets used and/or analysed during the current study are available from the corresponding author on reasonable request. Due to our General Data Protection Regulation (Art.5 DSGVO) we are not allowed to share sensitive data within an open data sharing platform. The results used here for purposes of statistical validation are based upon data generated by the TCGA Research Network: http://cancergenome.nih.gov/.

## Abbreviations

ccRCC: Clear cell renal cell carcinoma
CI: Confidence interval
HCC: Hepatocellular carcinoma
HIF: Hypoxia-inducible factor
HNSCC: Head and neck squamous cell cancer
IGF: Insulin-like growth factor
KIRC: Kidney renal clear cell carcinoma
OR: Odds ratio
OS: Overall survival
PIP2: Phosphatidylinositol 4,5-bisphosphate
PKC: Protein kinase C
qMSP: Quantitative methylation-specific polymerase chain reaction
RCC: Renal cell carcinoma
RFS: recurrence free survival
TACSTD2: Tumor Associated Calcium Signal Transducer 2
TCGA: The Cancer Genome Atlas network
VHL: Von Hippel–Lindau
