# Supplementary material for: DNA methylation of tumor associated calcium signal transducer 2 (TACSTD2) loci shows association with clinically aggressive renal cell cancers
Source: BMC Cancer. 2021 Apr 21;21:444. doi: 10.1186/s12885-021-08172-1 (PMC8061065; doi:10.1186/s12885-021-08172-1)
Supplement: Supplementary file 1 — Additional file 1: Suppl. Fig. 1: Correlation between TACSTD2 methylation and TACSTD2 mRNA expression. In silico analysis by Pearson correlation analysis reveal a statistically significant (p < 0.05) inverse relationship between TACSTD2 methylation (MethylVal) and TACSTD2 mRNA expression (Expr) in all investigated TACSTD2 loci. The Correlation coefficient (R) and the p-value are specified for each locus. [file 12885_2021_8172_MOESM1_ESM.pdf]

DNA methylation of Tumor Associated Calcium Signal Transducer 2 (*TACSTD2*) loci shows association with clinically aggressive renal cell cancers

Olga Katzendorn[1°], Inga Peters[1°*], Natalia Dubrowinskaja[1], Hossein Tezval[1], Pouriya Faraj Tabrizi[1], Christoph A. von Klot[1], Jörg Hennenlotter[2], Marcel Lafos[3], Markus A. Kuczyk[1], Jürgen Serth[1]

[1]Department of Urology and Urologic Oncology, Hannover Medical School, Hannover, Germany

[2]Department of Urology, Eberhard Karls University of Tuebingen, Tuebingen, Germany

[3]Department of Pathology, Hannover Medical School, Hannover, Germany

Running title: Prognostic significance of TACSTD2 DNA methylation in RCC

Key words: TACSTD2, DNA methylation, renal cell carcinoma, prognosis, survival

Conflict of Interest

All authors declare that they have no conflict of interest.
° Authors contributed equally to this work

***Correspondence to:**

Inga Peters

Carl-Neuberg-Str. 1

Department of Urology and Urologic Oncology,

Hannover Medical School,

30625 Hannover, Germany

Peters.Inga@mh-hannover.de

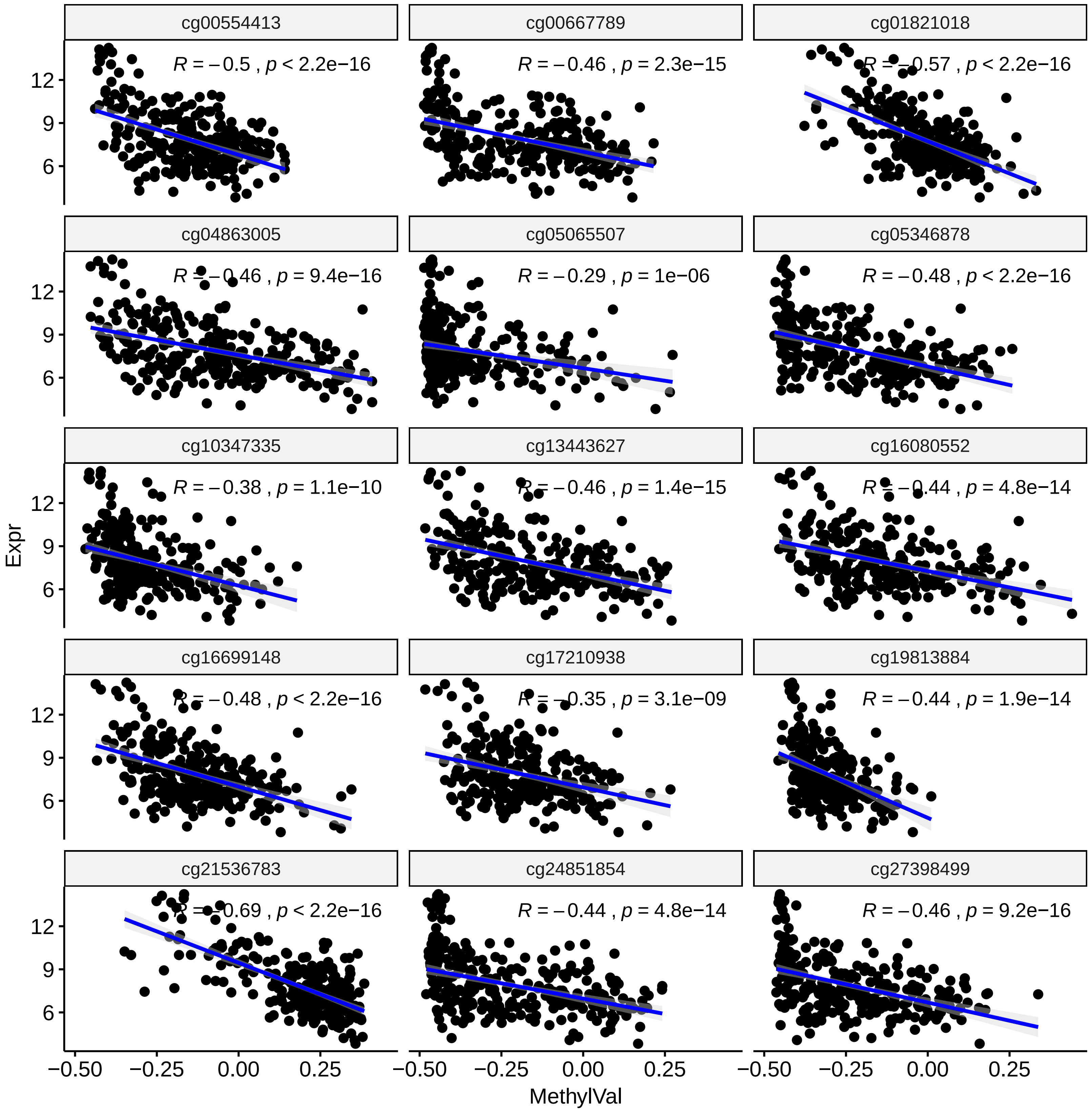
cg00554413
R = −0.5 , p < 2.2e−16
cg00667789
R = −0.46 , p = 2.3e−15
cg01821018
R = −0.57 , p < 2.2e−16
cg04863005
R = −0.46 , p = 9.4e−16
cg05065507
R = −0.29 , p = 1e−06
cg05346878
R = −0.48 , p < 2.2e−16
cg10347335
R = −0.38 , p = 1.1e−10
cg13443627
R = −0.46 , p = 1.4e−15
cg16080552
R = −0.44 , p = 4.8e−14
cg16699148
R = −0.48 , p < 2.2e−16
cg17210938
R = −0.35 , p = 3.1e−09
cg19813884
R = −0.44 , p = 1.9e−14
cg21536783
R = −0.69 , p < 2.2e−16
cg24851854
R = −0.44 , p = 4.8e−14
cg27398499
R = −0.46 , p = 9.2e−16
Expr
12
9
6
−0.50
−0.25
0.00
0.25
MethylVal
