# Supplementary figures and images for: DNA methylation of tumor associated calcium signal transducer 2 (TACSTD2) loci shows association with clinically aggressive renal cell cancers
Source: BMC Cancer. 2021 Apr 21;21:444. doi: 10.1186/s12885-021-08172-1 (PMC8061065; doi:10.1186/s12885-021-08172-1)

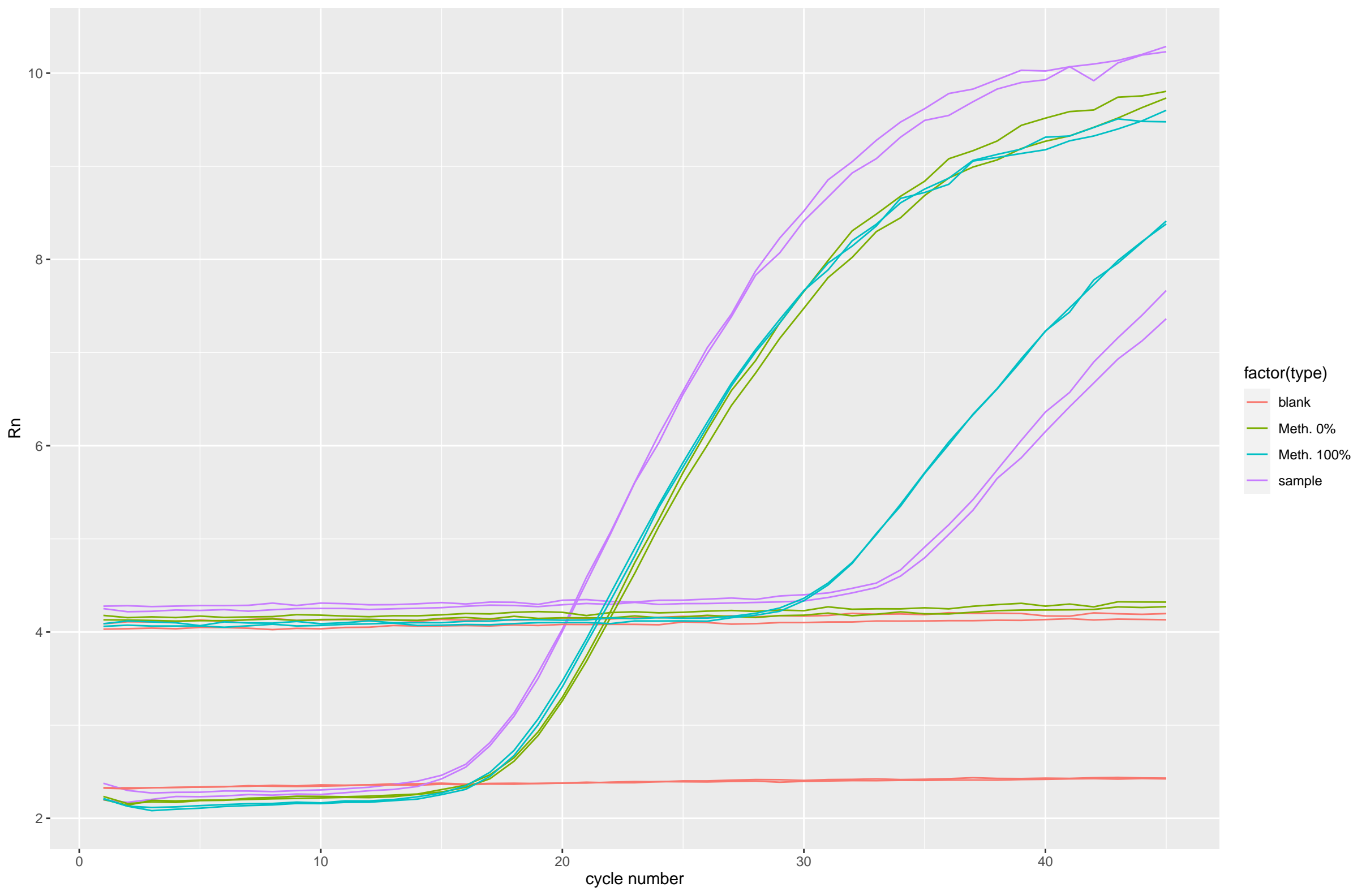

Supplement: Supplementary file 2 — Additional file 2: Suppl. Fig. 2: Exemplary primary data of quantitative methylation specific real-time PCR (qMSP). Sixteen measurements are required for determination and quality control of a single sample methylation. Measurements, presented without base-line adjustement, were carried out using either the Alu-C4 probe for adjusting for the input amount of converted DNA in blank, 0% methylation, 100% methylation and sample measurements each in duplicate (Group A) or aliquots of samples for corresponding measurements by use of the TACSTD2 probe (Group B). Note, that Alu-C4 detects a repetitive sequence motif resulting in considerable lower Ct values as compared to the single copy target sequence detection. [file 12885_2021_8172_MOESM2_ESM.pdf]
